# Supplementary material for: Long-term coding of personal and universal associations underlying the memory web in the human brain
Source: Nat Commun. 2016 Nov 15;7:13408. doi: 10.1038/ncomms13408 (PMC5116073; doi:10.1038/ncomms13408)
Supplement: Supplementary Information — Supplementary Figures 1-7 and Supplementary Tables 1-2. [file ncomms13408-s1.pdf]

Supplementary Information

Supplementary Figures

Supplementary Figure 1

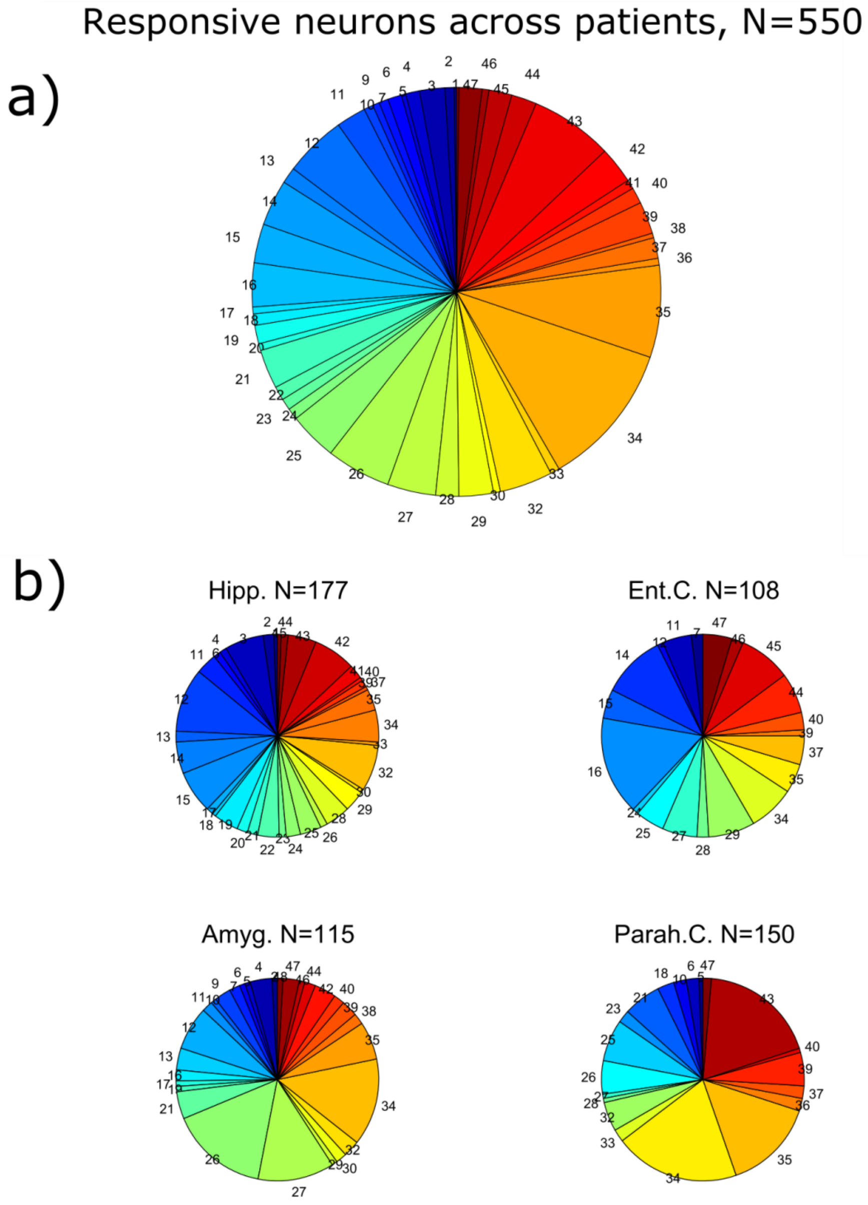

Supplementary Figure 1. a) Distribution of responsive neurons (Table S1, row 4) across patients. b) Distribution of responsive neurons per MTL area across patients. The numbers around the pie charts identify the patients.

Supplementary Figure 2

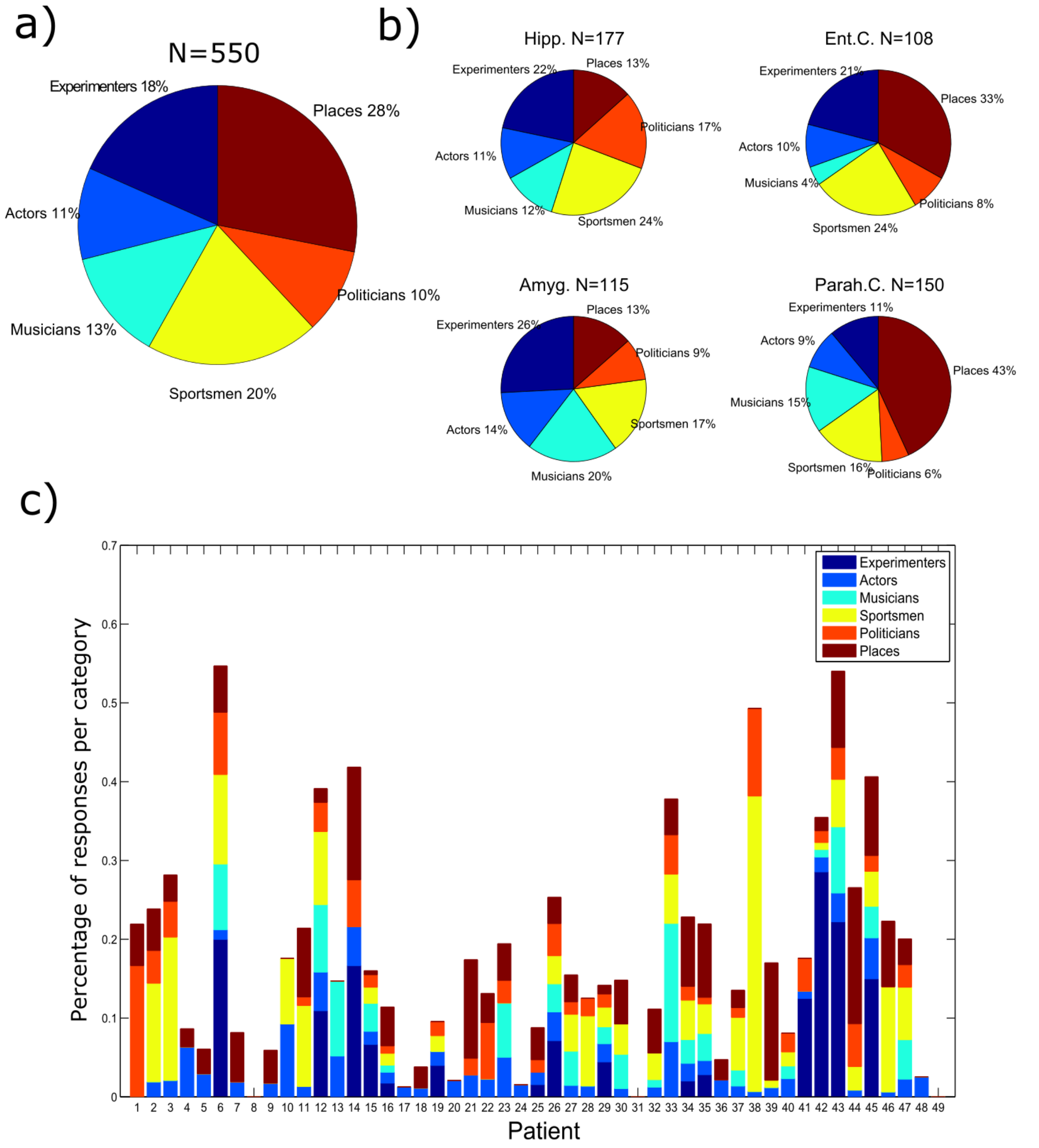

Supplementary Figure 2. Proportion of responses to each category for all responsive neurons (a) and for each MTL area (b). c) Percentage of responses per category for each patient. The percentage of responses for each category is plotted with stacked bars only for space reasons. Note, however, that it was calculated separately for each category (number of responsive stimuli divided by the number of stimuli presented for that category).

Supplementary Figure 3

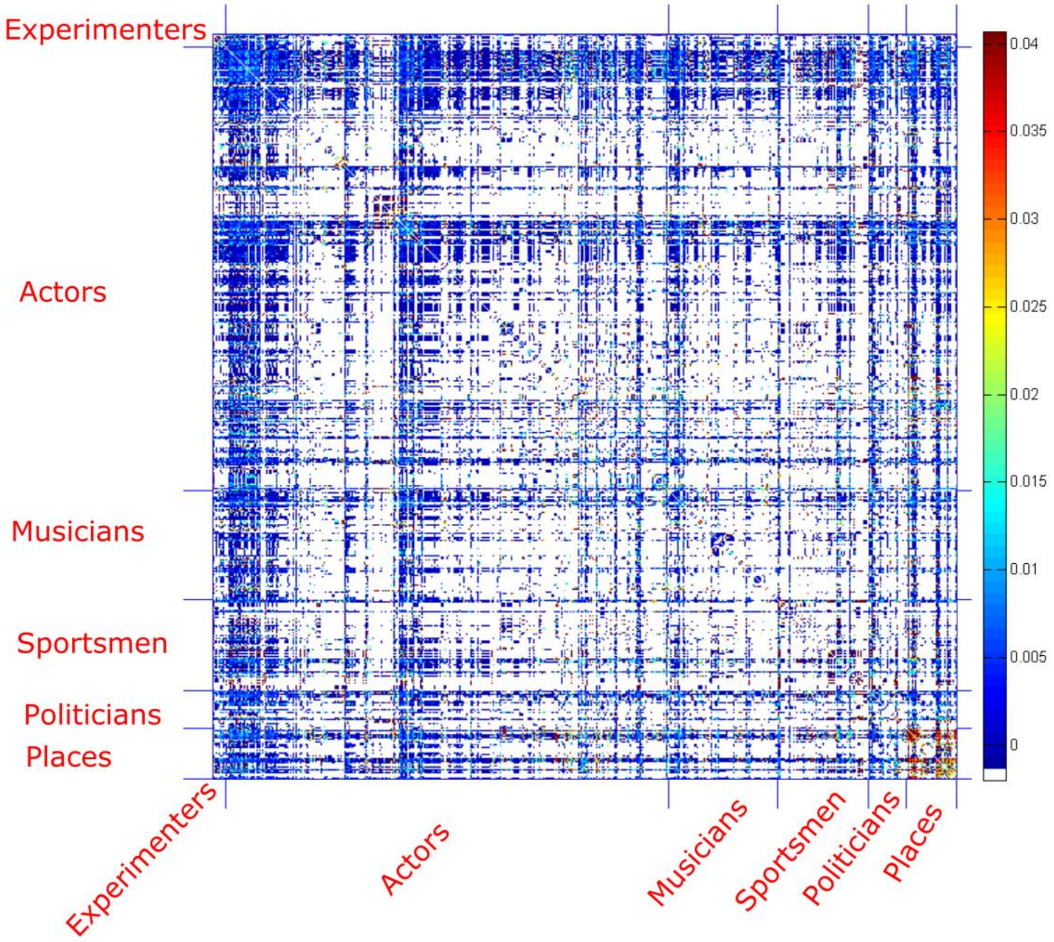

Supplementary Figure 3. Average joint-neural activation map. The white points in the map are empty entries in the matrix corresponding to pairs of stimuli that where never tested together.

Supplementary Figure 4

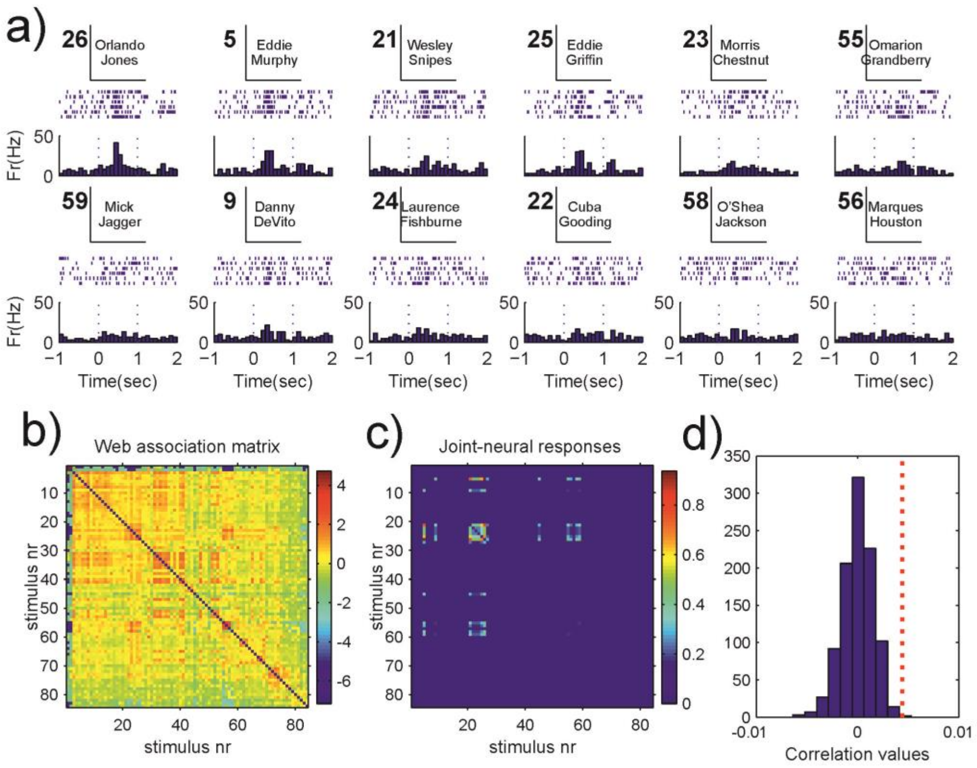

Supplementary Figure 4. A single neuron in entorhinal cortex that fired to pictures of several actors that are, according to the web-metric, related to each other's. Two of them (stimuli 25 and 26) were co-star in the movie "Double take". (b-c) Web-association and joint-response matrices for this neuron. Colorbars on the right denote association and join-response strength, respectively, in arbitrary units. (d) Correlation between the web-association and joint-response matrices (red dotted line), which was significantly larger than the values for a distribution of 1000 surrogates ($p<0.005$,rank test, 1000 surrogates). Owing to copyright issues, the images depicting people are replaced by their names. For the original figures see: https://www2.le.ac.uk/centres/csn/publications-1/2016/longtermcoding..

Supplementary Figure 5

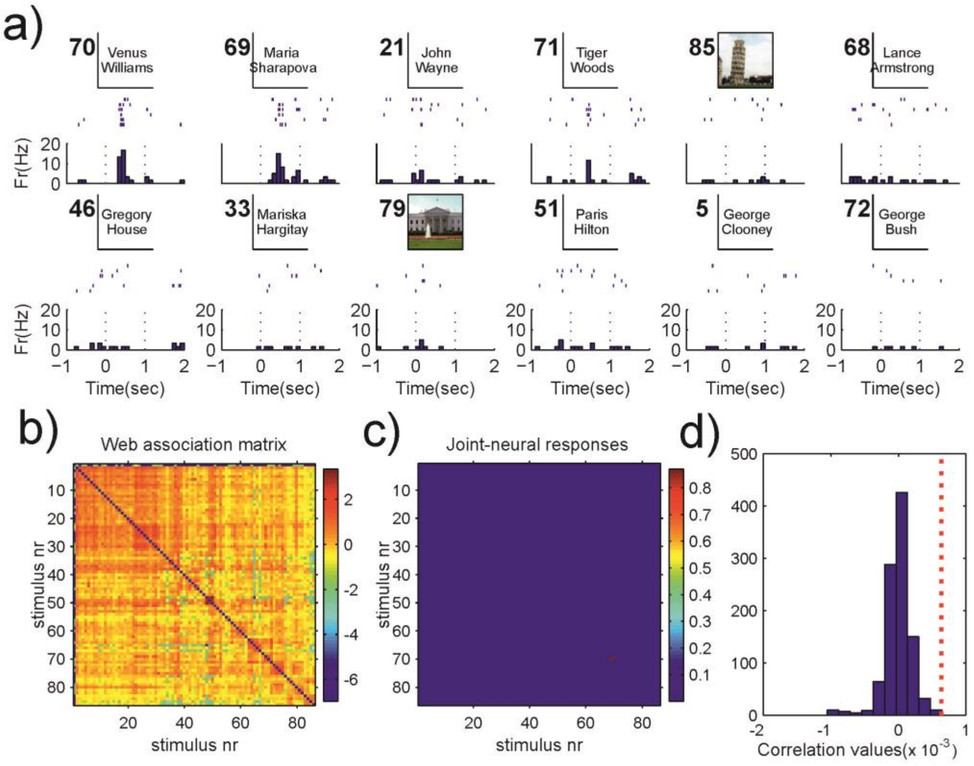

Supplementary Figure 5. A single neuron in amygdala that fired to pictures of two female tennis players (Venus Williams and Maria Sharapova). Conventions are the same as for Supplementary Figure 4. In this case, the correlation between the web-association and the joint-response matrices was significantly larger than chance with $p<0.01$ (rank test, 1000 surrogates).

Supplementary Figure 6

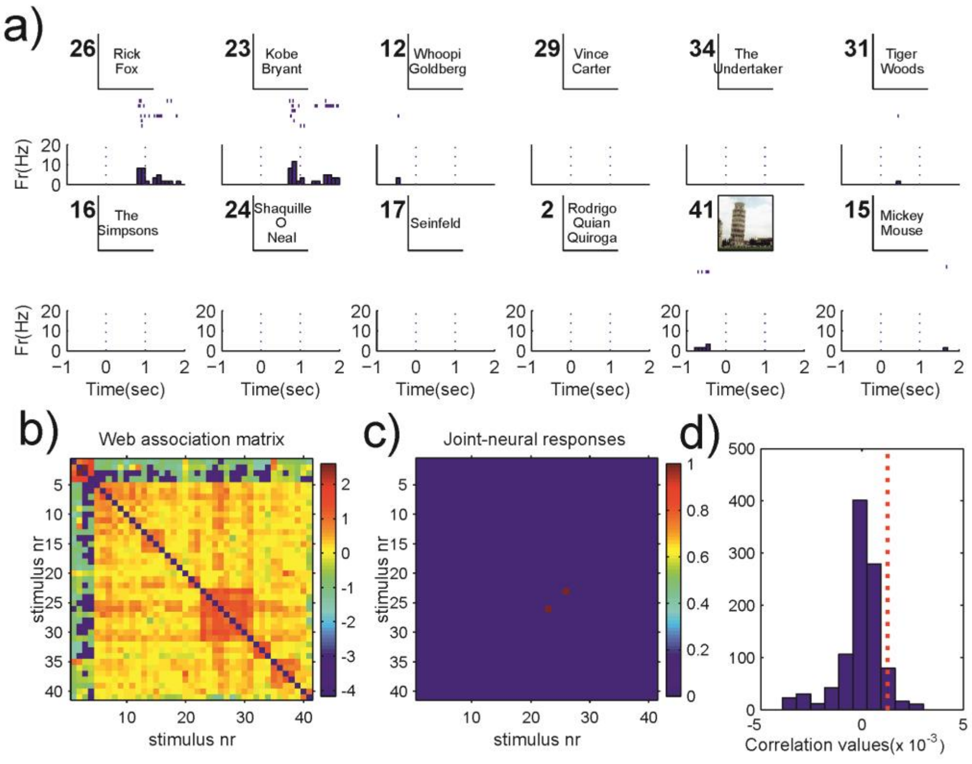

Supplementary Figure 6. A single neuron in the hippocampus that fired to pictures of two Los Angeles Lakers players: Rick Fox and Kobe Bryant. Conventions are the same as for Supplementary Figure 4. In this case, the correlation between the web-association and the joint-response matrices was significantly larger than chance with p<0.05 (rank test, 1000 surrogates).

Supplementary Figure 7

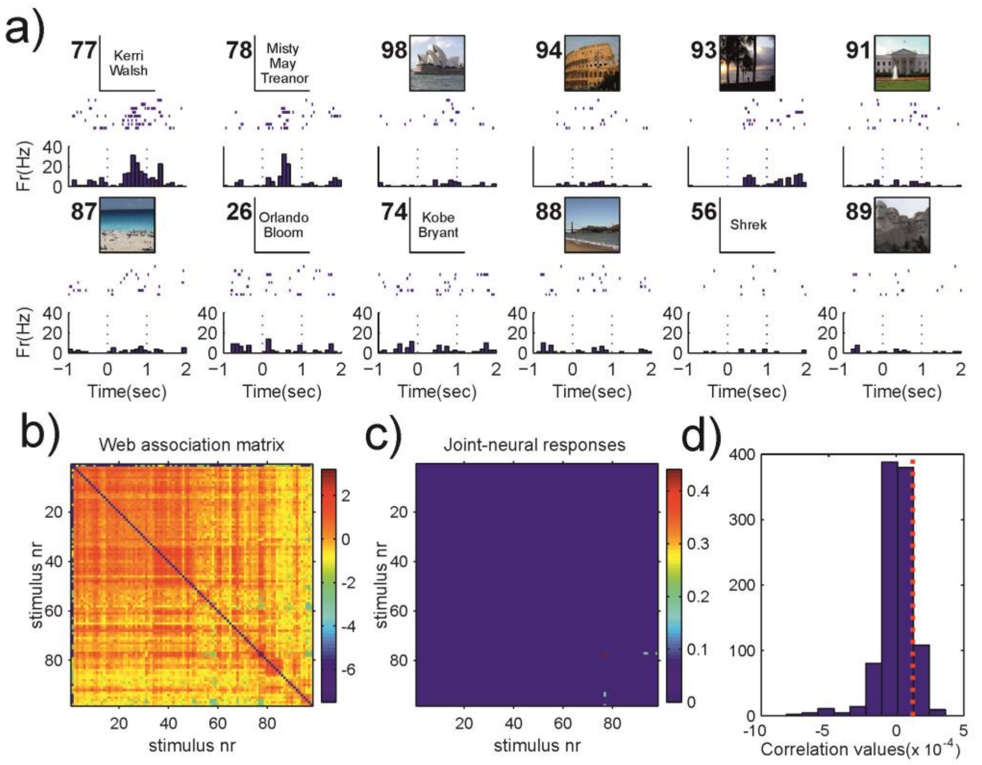

Supplementary Figure 7. A single neuron in the hippocampus that fired to the pictures of two beach-volleyball players, Misty May-Treanor and Kerry Walsh, who were teammates for several years. Conventions are the same as for Supplementary Figure 4. As for the previous example, the correlation between the web-association and the joint-response matrices was not significant (p=0.15, rank test, 1000 surrogates) given the relatively few associations encoded by the neuron.

Supplementary Tables

Supplementary Table 1

	Units	Single-units	Multi-units
Total number of recorded units	4078	1648	2430
Number of units with more than 1 response for which we had the patient's association scores (Figure 3a).	32	19	13
Number of units with more than 1 response for which we used the web-based metric scores (Figure 3b and 3d).	261	129	132
Number of responsive units (with 1 response or more) used to study the probability of pair responses (Figure 3e). The same units were used for the study of topographic representation (Figure 3c).	550	260	290
Number of units with a non-zero joint-neural response matrix used for the cell-by-cell correlation analysis (equation 4; see Figure 4d).	399	174	225
Number of units with at least two non-zero values in the upper half of the joint-neural response matrix used for the decoding analysis (equation 5).	345	136	209

Supplementary Table 1. Number of units used for the different analyses.

Supplementary Table 2

	Recorded Units		Responsive Units		Multi-responsive Units	
	SU	MU	SU	MU	SU	MU
Hippocampus	480	711	92	85	42	30
Entorhinal cortex	383	534	59	49	33	23
Amygdala	558	737	62	53	24	22
Parahippocampal cortex	227	448	47	103	30	57
Total	1648	2430	260	290	129	132

Supplementary Table 2. Number of single-units (SU) and multiunits (MU) recorded (corresponding to Supplementary Table 1, row 1), number of responsive units (Supplementary Table 1, row 4) and of multiresponsive units (Supplementary Table 1, row 3) per MTL area.
